# Pulsed field ablation: from concepts to technology

**DOI:** 10.1093/europace/euag205

**Published:** 2026-10-21

**Authors:** Ante Anić, Antoni Ivorra, Brendan Koop, Nathan J Kirkpatrick, Jacob Koruth

**Affiliations:** Department of Cardiovascular Disease, University Clinical Hospital, Split, Croatia; Department of Engineering, Universitat Pompeu Fabra, Barcelona, Spain; Department of Electrophysiology, Boston Scientific Corporation, 4100 Hamline Ave. N, St. Paul, MN 55112, USA; Department of Electrophysiology, Boston Scientific Corporation, 4100 Hamline Ave. N, St. Paul, MN 55112, USA; Helmsley Electrophysiology Center, Mount Sinai Fuster Heart Hospital, New York, NY, USA

**Keywords:** Pulsed field ablation, Irreversible electroporation, Cardiac arrhythmias, Electric field, Waveform design, Catheter design, Computational modeling

## Abstract

**Aims:**

Pulsed field ablation (PFA) has emerged as a transformative approach for treating cardiac arrhythmias by exploiting irreversible electroporation to preferentially injure myocardium while limiting collateral damage. Translating this biophysical principle into safe and effective clinical systems requires careful integration of waveform design, catheter engineering, computational and experimental modelling, safety testing, and procedural workflow.

**Objective:**

This review summarizes the fundamental physical and engineering principles that govern cardiac PFA and examines how these principles influence efficacy, safety, lesion formation, and clinical usability.

**Biophysical and technical considerations:**

Electroporation is governed primarily by the local electric field and waveform-dependent lethal thresholds rather than generator voltage alone. Key waveform variables include monophasic vs. biphasic pulses, microsecond vs. nanosecond pulse widths, duty cycle, and total application duration. Catheter design further determines electric field distribution, lesion geometry, current density, and applicability across anatomic targets, with important distinctions between bipolar and monopolar configurations.

**Modelling and safety:**

Experimental and computational approaches used to investigate PFA include finite element simulations, cell culture systems, vegetal substrates, and *in vivo* animal models, each with distinct strengths and limitations. High-voltage pulse delivery can also produce unintended effects, including Joule heating, electrochemical reactions, bubble formation, arcing, and interactions with implanted electronic devices. These phenomena highlight the importance of optimized waveform design, electrode configuration, and safety monitoring.

**Clinical usability:**

Clinical adoption depends not only on biological efficacy and safety but also on workflow efficiency, catheter versatility, procedure duration, mapping integration, anaesthesia requirements, and adaptability to differing clinical practices and geographic settings.

**Conclusions:**

The performance of a PFA system reflects the interaction of biophysics, waveform characteristics, catheter design, modelling, safety controls, and procedural usability. Understanding these interconnected principles is essential for developing PFA technologies that are effective, safe, predictable, and practical for contemporary electrophysiology laboratories.

## Graphical Abstract

**Graphical Abstract:**
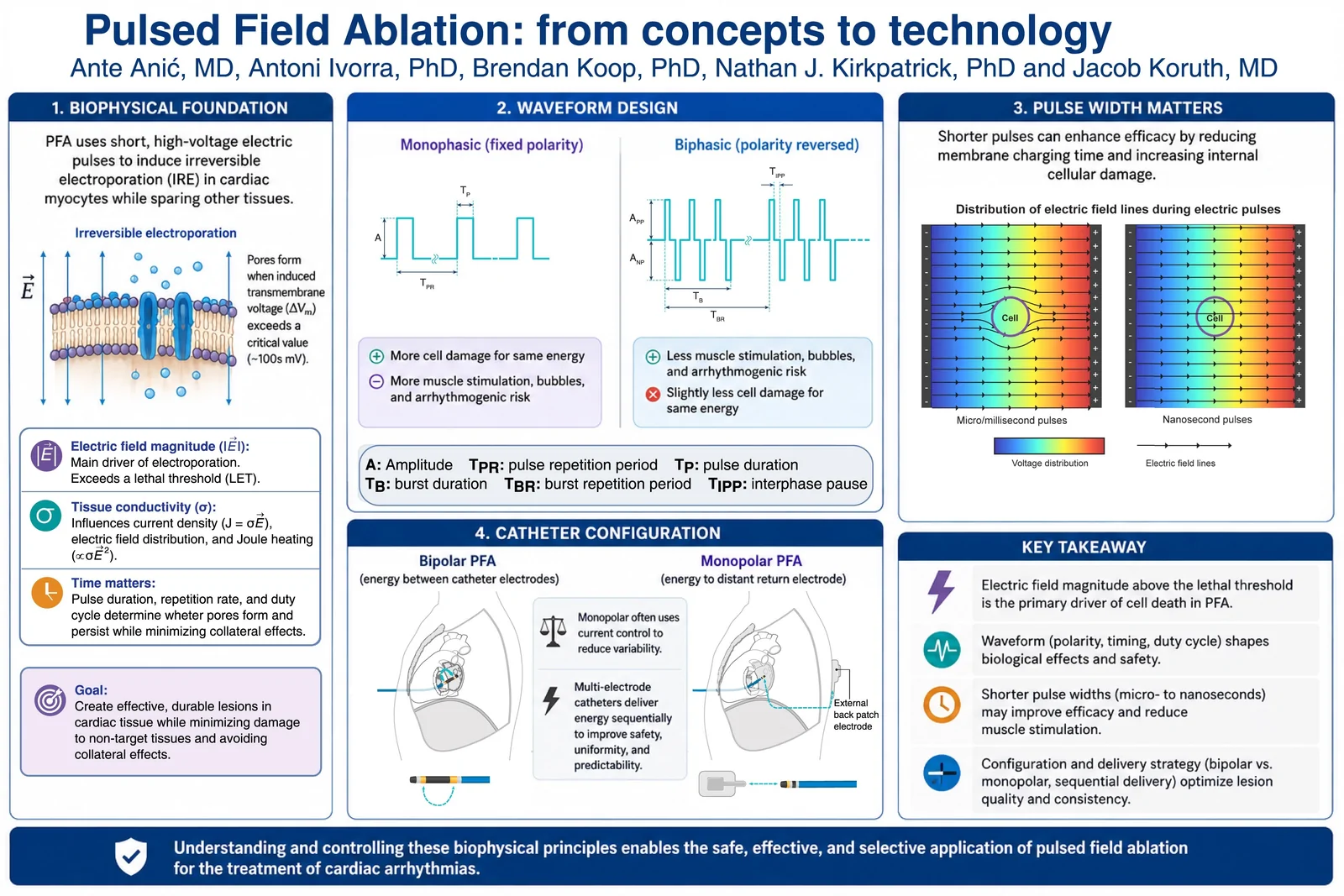

## Introduction

Pulsed field ablation (PFA) to treat cardiac arrhythmias is a revolution in the ability to preferentially ablate myocardial cells, with several advantages over thermal ablation technologies. However, it is a large leap from the concept of PFA to the actual implementation of technologies that allow this revolution to proceed. The aim of this article is to describe how the biophysical principles of PFA can be harnessed in safe, effective, and usable technical implementations.

## Main physical variables in pulsed field ablation

The cytotoxic effect of PFA is driven by irreversible electroporation (IRE), and the two terms are often regarded as synonymous.^1^ Much of the current understanding of PFA for treating cardiac arrhythmias originates from earlier use of IRE to treat solid tumours.^2^ Therefore, the biophysical foundations of PFA are those of electroporation.

Electroporation refers to the increase in cell membrane permeability to ions and molecules in response to a sufficiently strong electric field.^3^ This phenomenon is commonly approximated as a threshold process: it occurs when the change in transmembrane voltage $\left(\Delta V_{m}\right)$ induced by the applied electric field $\left(\vec{E}\right)$ exceeds a critical value on the order of hundreds of millivolts.

The induced transmembrane voltage is directly proportional to the magnitude $\left(|\vec{E}|\right)$ of the applied electric field, commonly expressed in units of V/cm. For spherical or ellipsoidal cells in suspension, simple analytical expressions can be derived to relate the applied field to the induced transmembrane voltage.^4^ Although simulations have been performed using idealized tissue structures,^5,6^ accurately predicting induced transmembrane voltages in real tissues remains infeasible due to insufficient knowledge of their detailed architecture. Nevertheless, because the proportionality between the induced transmembrane voltage and the electric field is preserved, it can be assumed that electroporation occurs when the local electric field magnitude exceeds a critical threshold.

While the main physical variable that drives electroporation in PFA is the local electric field magnitude, the parameter that is usually reported or can sometimes be modified by the operator in PFA generators is the output voltage. It is important to understand that output voltage and electric field magnitude are not equivalent. The application of voltage by a PFA generator creates an electric field distribution between the electrodes. However, the magnitude of this electric field depends not just on the voltage delivered but also on the geometry of the catheter.

Electroporation is not a static phenomenon but a dynamic process, and the temporal characteristics of the applied field also play a crucial role in PFA. According to molecular dynamics simulations,^7,8^ pores form in the cell membrane within nanoseconds after the critical transmembrane voltage is reached. However, this critical voltage is not attained immediately and requires several fractions of a microsecond, primarily depending on cell size. The lipid bilayer acts as an insulating barrier between two conductive media (the intracellular and extracellular ionic solutions) and therefore behaves like a capacitor. Mobile ions in the surrounding medium must accumulate across the membrane to establish the transmembrane voltage. Consequently, when high-frequency alternating fields are applied, the membrane may repeatedly charge and discharge without reaching the critical voltage necessary for electroporation.^9^  *Table 1* provides definitions for basic principles associated with PFA.

**Table 1:** Basic physical parameters relevant in PFA

Parameter	Common symbols	Description	Implications
Induced transmembrane voltage	$\Delta V_{m}$	Change in transmembrane voltage caused by the presence of an electric field, expressed in volts (V)	Electroporation occurs when $\Delta V_{m}$ exceeds a critical value, on the order of hundreds of millivolts.
Electric field	$\vec{E}$	At each point in space, a vector quantity (i.e. it has magnitude and direction) that represents the electric force that a charge of 1 coulomb would experience if placed there	The electric field arises from the presence of neighbouring charges, each of which would exert a force on the test charge according to Coulomb’s law. Under equilibrium conditions, positive and negative charges in biological tissues are balanced and evenly distributed, resulting in a null macroscopic electric field. When a voltage is applied across electrodes or a current is driven through the tissue, this balance is disrupted, and an electric field is induced.
Electric field magnitude	$\left|\vec{E}\right|$ or, simply, E	Magnitude of the electric field vector, expressed in V/m in SI units, but is often reported in V/cm	In electroporation treatments, the electric field magnitude is not constant but takes the form of a time-varying waveform. For a specific tissue and a given waveform, there exists a critical electric field magnitude that must be exceeded to achieve the intended electroporation effect (e.g. molecular uptake or tissue ablation). The critical field threshold decreases as the total energy of the waveform increases. However, it is important to note that this dependence cannot be described with a linear relationship.
Lethal electric field threshold	LET	Magnitude of the electric field vector required to ablate a specific tissue for a given waveform.	Very few published *in vivo* studies report lethal electric field thresholds in the heart while also fully disclosing the applied waveform: 360 V/cm (pig, 48 monophasic pulses of 100 µs),^10^ 640 V/cm (rat; 10 monophasic pulses of 100 µs) and ∼1200 V/cm (rat, 10 bursts of 10 biphasic pulses of 5 µs+5 µs).^11^ Commercial PFA systems likely employ waveforms that produce cardiac lethal electric field thresholds in the range of 300–700 V/cm.^12^
Tissue conductivity	*σ*	Quantifies how easily electric charges can move through a material under an applied electric field, expressed in siemens per metre (S/m)	It is determined by both the concentration of free charge carriers and their mobility within the medium. In soft living tissues, it typically ranges from ∼0.02 S/m (fat) to 1.5 S/m (plasma). Electroporation can cause a substantial increase in tissue conductivity. Tissue conductivity also increases with temperature (∼2%/°C).^13,14^ In anisotropic materials, conductivity is not a single value but depends on the direction of current flow. This is observed in skeletal muscle where the longitudinal conductivity ($\left(\sigma_{L}\right)$)—aligned with the fibre orientation—is significantly higher than the transverse conductivity ($\left(\sigma_{T}\right)$).
Applied voltage	*v*	Applied voltage across electrodes, expressed in volts (V)	The amplitude of the waveforms applied in electroporation treatments, including cardiac PFA, typically ranges from several hundred volts to a few kilovolts.
Delivered current	*i*	Resulting current through tissues when a voltage is applied across electrodes, expressed in amperes (A)	The relationship between voltage and current is defined by Ohm’s law: v=iR where R is the resistance of the conductor, expressed in ohms (Ω). The resistance depends on the conductivity of the material but also on its geometry (i.e. the electrodes geometry and their distance). Current amplitude in electroporation treatments typically ranges from several amperes to a few tens of amperes.
Current density	$\vec{J}$	Vector quantity that represents both the magnitude and direction of electric current flow per unit area	According to Ohm’s law, $\vec{J}=\sigma \vec{E}$
Current density magnitude	$\left|\vec{J}\right|$ or, simply, J	Magnitude of the current density vector, expressed in A/m^2^	$\left|\vec{J}|=\sigma |\vec{E}\right|$
Joule heating	$Q_{J}$	Heat produced due to the flow of electric current in a conductive material, typically expressed as power density, that is, as watts per unit volume (W/m^3^)	It is proportional to the square of the electric field magnitude $\left(Q_{J}=\sigma {\left|\vec{E}\right|}^{2}\right)$. This implies that in regions where the electric field is highly concentrated—such as near the sharp edges of electrodes—thermal energy dissipation may increase dramatically. As a reference for its impact: a single pulse of 100 µs with an amplitude of 1000 V/cm will produce a sudden temperature increase of about 0.35°C in saline. The accumulative temperature increase of multiple exposures can be counteracted by heat dissipation mechanisms such as blood flow. However, it must be noted that while the temperature increase is instantaneous, heat dissipation may take several seconds or even minutes to return the tissue to baseline temperature. Exposure to 50°C for several seconds causes damage to all soft tissues.

## Models to study electroporation

This section describes the experimental models commonly used to study electroporation, ranging from *in silico* to *in vivo* models at different complexity levels. As the mechanisms of PFA are fundamentally different from other thermal ablation technologies, the models used to study this phenomenon are also conceptually different.

### Computational models

#### Basic concepts

As explained above, the main physical parameter governing PFA is the electric field. Therefore, accurate calculations of the electric field distribution for a specific geometry are fundamental to the study of a PFA system. Most electroporation computer models at the tissue scale operate under the assumption that, for a specific tissue and a given waveform, there exists a critical electric field magnitude that must be exceeded to achieve the intended electroporation effect. Importantly, this critical field magnitude—such as the lethal threshold for cardiomyocytes in cardiac PFA—is not only tissue-specific but also waveform-specific. Consequently, it is not straightforward to scale these thresholds across different waveforms. Unlike thermal damage, where a metric can be computed and used to extrapolate thresholds, no analogous dosimetric metric exists for electroporation. This means that thresholds must be determined experimentally for each distinct waveform. However, it is worth noting that US Food and Drug Administration researchers have recently published comprehensive *in vitro* data characterizing a wide range of PFA waveforms, which can serve as a reference for approximating how thresholds vary with waveform features such as pulse duration and number.^15^

While analytical expressions can be derived to compute electric field distributions for idealized electrode geometries in homogeneous tissues, these models rarely reflect clinical reality. In practice, electrode configurations are often complex, and biological tissues exhibit highly irregular geometries and heterogeneous electrical properties. Consequently, numerical techniques—and in particular the finite element method—are used to accurately map the electric field distribution and predict which regions will reach the necessary thresholds for successful electroporation.^16^

Zooming out in both time and spatial scale, a variety of models based on different mechanistic or phenomenological assumptions have been proposed to describe membrane permeabilization to ions and molecules over time scales on the order of seconds to minutes.^17^ However, none of these models have been able to consistently reproduce the full range of experimental observations.

#### Computational modelling of lesion formation in pulsed field ablation

The validity of computational modelling of electroporation treatments based on the assumption of a critical electric field threshold has been demonstrated repeatedly across a range of scenarios. This approach has been validated *in vitro*^18^ in relatively homogeneous tissues such as plant tissues^19^ and liver (*in vivo*^20^ and clinically,^21^), as well as in some heterogeneous scenarios that involve tailored treatment planning.^22,23^ Crucially, the accuracy of this modelling approach has improved with the incorporation of tissue conductivity dependence on both the electric field—where conductivity increases due to electroporation—and temperature, which rises because of Joule heating.^24^ Hence, it is unsurprising that published cardiac PFA studies performing computer modelling for lesion formation adopt this framework.^10–14,25–30^

It should be noted that careful histological observations, particularly of lesions created with monopolar catheters, have shown that the conventional modelling framework cannot accurately predict the shape of actual cardiac PFA lesions.^31,32^ Ventricular myocardial lesions are substantially deeper than predicted by the models. Even after accounting for conductivity anisotropy, lesions are still not accurately represented, and in some cases, anisotropy predicts shallower lesions. Lesion dimensions can be reasonably approximated if two distinct thresholds are used for orthogonal fibre orientations.^33^ Nevertheless, the underlying cause of this behaviour, which had not been reported previously in the electroporation community, remains unclear. One hypothesis is that the effect arises from anisotropic tissue damage, with cardiomyocytes oriented transverse to the electric field being more susceptible due to a larger exposed membrane area. A computational multiscale model has been developed that appears to support this hypothesis and suggests that this mechanism may partially explain the observed lesion morphology.^34^

### *In vitro* models

In vitro models have been essential for studying the electroporation phenomenon. Since electroporation is a universal phenomenon possible in any lipid bilayer, highly idealized *in vitro* models—specifically planar artificial membranes (black lipid membranes) and giant unilamellar vesicles—have been utilized to elucidate the physical and biochemical fundamentals of electropermeabilization.^35,36^

Cell cultures have provided further insights into the biological consequences of electroporation and how these effects are modulated by diverse physiological variables. Notable efforts have involved the use of adult cardiomyocytes isolated from living tissue,^37,38^ rat ventricular cardiomyocytes,^33,34^ immortalized cell lines,^39^ and human induced pluripotent stem cell-derived cardiomyocytes.^40^ Cell culture studies have highlighted the importance of repeated pulses for effective permeabilization^41^ and have provided insight into the mechanisms of cell death following pulse exposure.^42^ These models have also demonstrated the critical role of extracellular calcium in determining cell death.^43–46^

It is worth highlighting the study by Maizels and colleagues, in which electrical activity in two-dimensional cultures of cardiomyocytes was subjected to different PFA protocols using voltage-sensitive fluorescent probes.^47^ Remarkably, this study demonstrated that PFA can terminate arrhythmias with high spatial precision by creating sustained bidirectional conduction block lines. However, it also revealed that the acute presence of functional conduction block does not necessarily translate into long-term lesion persistence, a well-recognized issue in clinical cardiac PFA.^28^

A limitation of conventional cell cultures is their inability to replicate the dense cell packaging found in living tissues. Consequently, these models fail to simulate the significant increase in bulk conductivity that occurs during electroporation—an effect that alters electric field distribution and subsequent lesion formation. This constraint, combined with a lack of three-dimensionality, renders standard cell cultures unsuitable for evaluating catheters. Cell spheroids address these limitations, yet they cannot reach large sizes without developing an ischaemic or necrotic core.^48^

### Vegetal models

Excised animal tissues can only be used for electroporation studies for a few hours after removal^49^ due to the rapid deterioration of their cell membrane integrity. In contrast, plant tissues exhibit greater structural resilience after excision, primarily due to the presence of rigid cell walls and a reduced reliance on immediate energy-dependent processes for maintaining cellular integrity. As a result, vegetal tissues, specifically potato tubers, are frequently utilized in electroporation studies. A distinct advantage of this model is that irreversibly electroporated regions naturally darken over several hours,^25^ which eliminates the need for staining, although staining can still be employed for faster and more precise visualization of the lesions.^50,51^

While potato tubers are often assumed to be homogeneous, they possess inherent structural variations, and their conductivity properties and lethal critical thresholds substantially differ from those of the myocardium. Nevertheless, despite these physiological discrepancies, they constitute an attractive intermediate model for minimizing the necessity of *in vivo* assays. In this regard, their use aligns with the Replacement principle of the 3Rs (Replacement, Reduction, and Refinement) ethical framework for animal research.^52^

### *In vivo* models

*In vivo* models constitute the most realistic framework for studying the acute and chronic effects of electroporation in human tissues.^27,30^ Preclinical porcine models are considered the gold standard due to their anatomical and electrophysiological similarities to the human heart.^53^ However, ovine models have also been used in preclinical studies due to their ability to withstand relatively long surgical procedures.^54^

Lesions are often assessed using imaging methods such as late gadolinium enhancement MRI. However, the most accurate assessment remains histopathology, which allows for a detailed cellular analysis of the ablation zone. In addition, triphenyl tetrazolium chloride (TTC) staining is frequently used because it provides a fast, macroscopic evaluation of tissue viability. By reacting with enzymes in healthy mitochondria, TTC stains viable tissue red while leaving lesions pale, allowing acute lesion volume to be clearly demarcated shortly after the procedure. Remarkably, TTC staining exhibits a hyperstained red zone around the lesion, which has been attributed to reversible electroporation.^55^ The timing between PFA treatment and lesion analysis influences results. Waiting at least 4 h after PFA allows reversible electroporation to recover and irreversible areas to undergo cell death. Delays of 2–7 days after the procedure may more accurately reflect chronic lesion size.^38^

## Physical effects of high electric fields

### Joule heating

While PFA has a non-thermal mechanism of action, introduction of high voltage across a resistive load such as myocardial tissue will inevitably result in some thermal impact. This effect, called Joule heating, is proportional to the conductivity of the tissue and scales with the square of the electric field. Unlike traditional radio frequency (RF) catheters, PFA involves energy delivery via pulse trains with pulse widths on the order of milliseconds to microseconds. This reduction in energy delivery time produces transient thermal spikes. Furthermore, the peak temperatures are found at the edges of the electrode surfaces, where current densities are the highest.^2^ Excessive Joule heating as a by-product of non-optimized PFA workflows can result in coagulative necrosis of tissue,^56^ and microbubble formation.^57^

A time-temperature history governs the generation of thermal injury. Arrhenius thermal damage models can be used to show the effects of cumulative thermal injury on tissue^58^ and the blood.^59^ Coagulative necrosis can occur when temperatures above 55°C are sustained for over 70 s^60^ timescales common for RF ablations (*Figure 1*). With the localized and pulsatile nature of Joule heating with PFA, peak temperatures are sustained for less time, allowing for the potential for higher maximum temperatures while still maintaining a less impactful time-temperature history. However, PFA waveform considerations are paramount to ensure that inter-pulse intervals are sufficient to allow for thermal dissipation between pulses^61^ while resulting in greater and more stable membrane permeabilization.^41,62^

**Figure 1:**
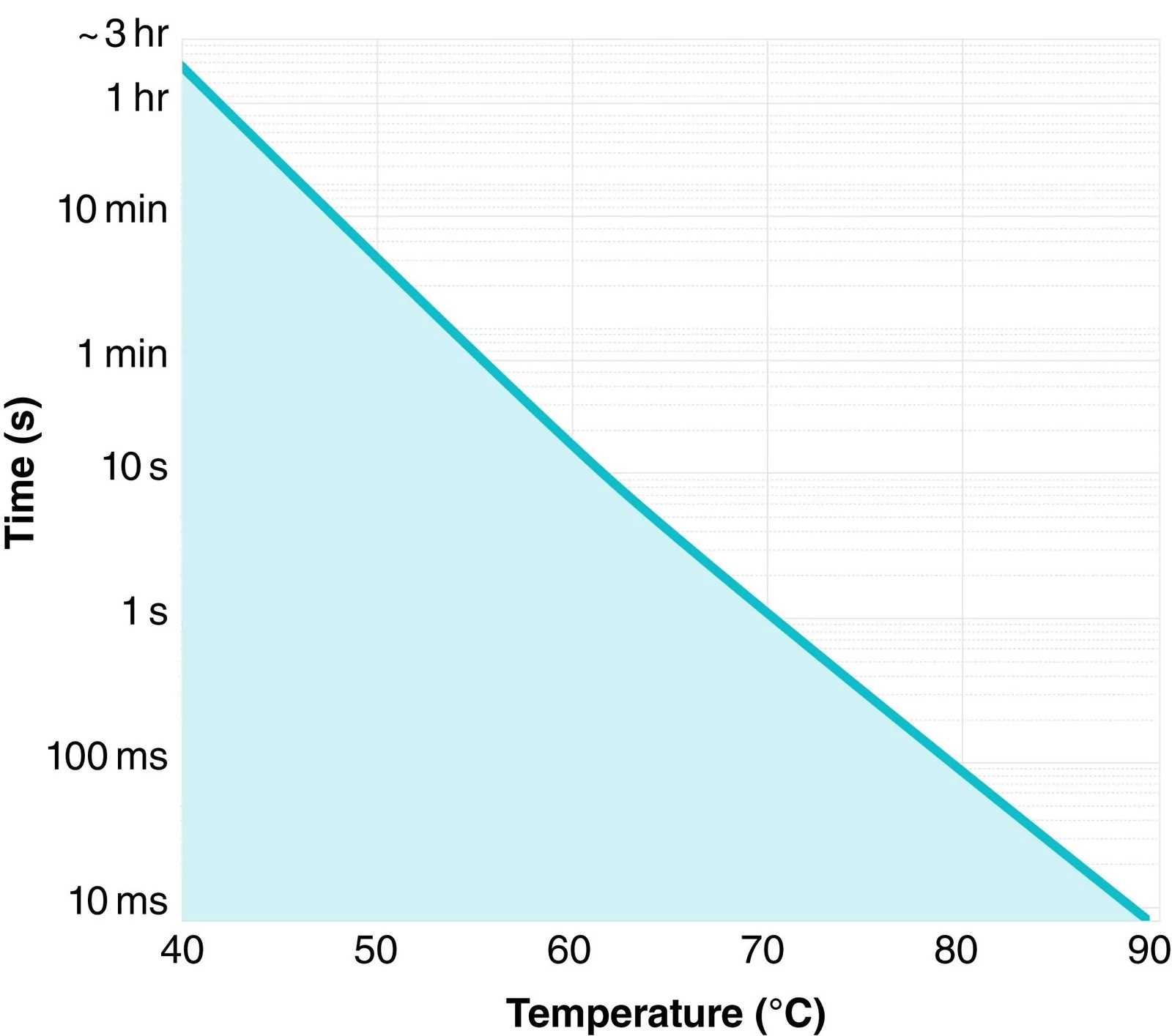
Time duration required to cause thermal damage at various temperatures. An Arrhenius-type model was fit to experimentally derived data using parameters for cardiac tissue previously published. Data taken from Niemiera, B., Ranjan, R., Piccini, J.P. et al. Microbubble and thermal profile of an eight-spline pulsed field ablation system in a preclinical model. J Interv Card Electrophysiol (2025). https://doi.org/10.1007/s10840-025-02145-1.

### Electrochemical reactions

Electric charge carriers in conventional electrical circuits are electrons, whereas in biological tissues, the charge is carried by ions (both anions and cations). Therefore, any system that connects electronic conductors to biological media must include interfaces where this change in charge carriers occurs. These interfaces are found at the electrodes.

Electrodes can enable the flow of electricity through two distinct mechanisms: capacitive (non-faradic) and electrochemical (faradic). In the capacitive mode, the interface between the conductive electrode and the ionic medium (i.e. the electrolyte) behaves as a capacitor. A layer of water molecules acts as a dielectric, physically separating the electrons in the metal from the ions in the electrolyte. Here, charges do not cross the interface; only accumulation and deaccumulation of charge occur. Consequently, only transient or time-varying currents can be sustained via this capacitive pathway.

Continuous currents, on the other hand, can flow if the electrochemical pathway is engaged. Here, electron exchange reactions (i.e. reduction and oxidation reactions) occur between the chemical species in the ionic medium and those of the electrode. For most metals used in catheters, such as gold, platinum, titanium, and stainless steel, these reactions do not occur spontaneously. Electrons require a small amount of energy, typically achieved by applying hundreds of millivolts to overcome the energy barrier and initiate the reactions. However, in electroporation treatments, where hundreds to thousands of volts are applied, electrochemical reactions at the electrodes become substantial. These reactions can generate pH gradients, release gaseous microbubbles, and liberate metallic ions, all of which pose a risk of localized tissue toxicity or, as discussed below, embolic events. *Figure 2* illustrates the electrochemical reactions expected at a pair of stainless steel electrodes in saline. While the electrolysis of water is almost always the dominant process, secondary reactions—such as the evolution of chlorine gas and the anodic dissolution of metal ions—frequently occur simultaneously.

**Figure 2:**
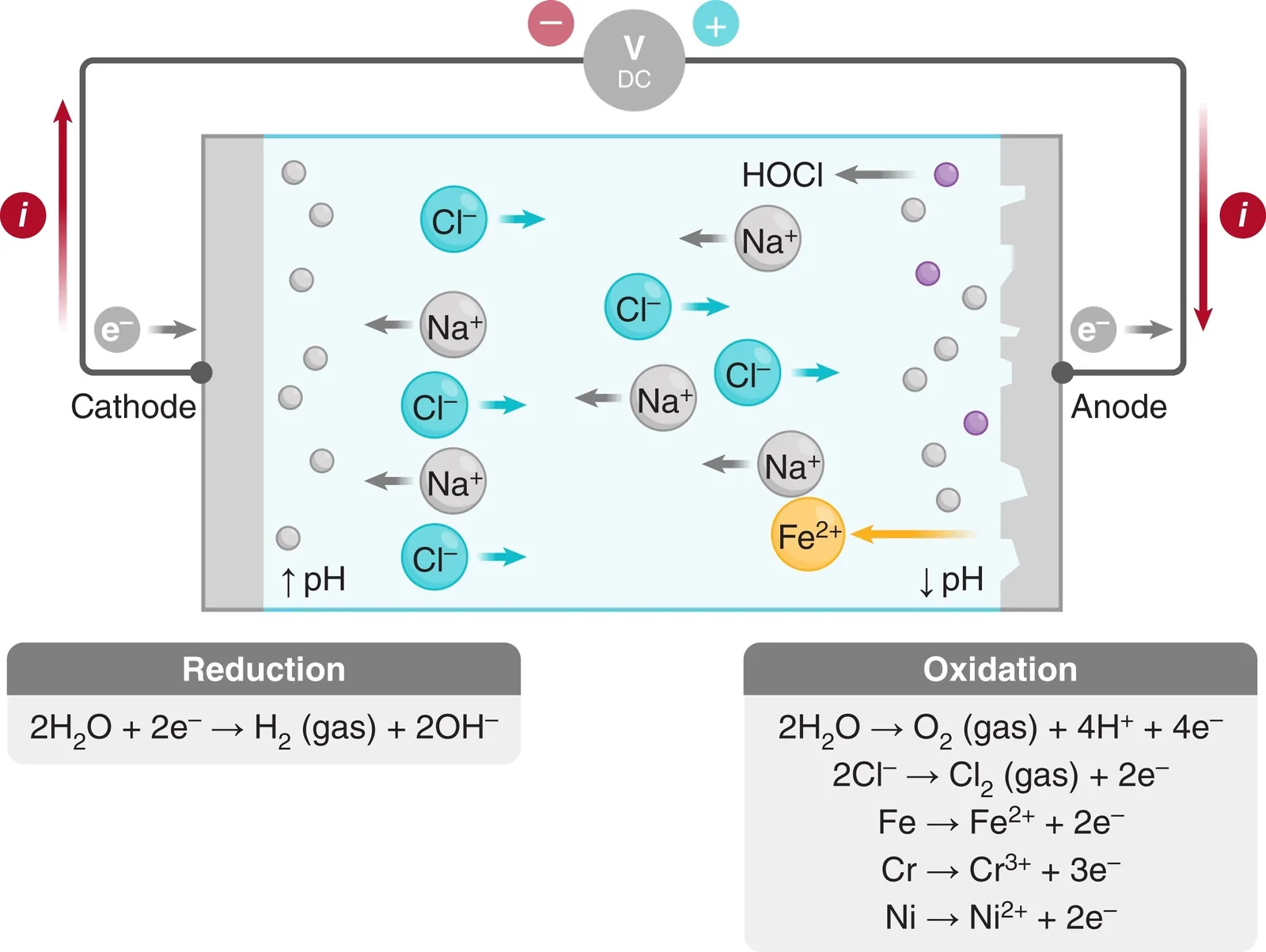
Electrochemical reactions due to a voltage between stainless steel electrodes in saline.

Nevertheless, it must be noted that when high-frequency biphasic waveforms are applied, the capacitive pathway becomes highly favoured. Due to the high rate of change in voltage, the displacement current across the dielectric interface dominates, effectively bypassing the slower faradaic processes. Furthermore, electrochemical products generated during a phase are often partially or fully reversed during the subsequent phase of opposite polarity. This rapid alternation prevents the accumulation of long-lived chemical species and gaseous precursors at the electrode surface, thereby significantly mitigating the adverse impacts of electrochemical reactions.

### Bubbles

When high current is forced through electrodes in an aqueous electrolyte—such as blood plasma or intra- and extracellular media—gas bubbles may form via three pathways: the previously discussed electrochemical reactions, the formation of steam bubbles due to Joule heating, and the release of dissolved gases (e.g. nitrogen) resulting from the inverse relationship between temperature and solubility. This topic has recently been addressed experimentally by Mahnič-Kalamiza and colleagues.^57^ In their *in vitro* saline study, they found that bubbles arising from electrochemical reactions are more prevalent under monophasic pulsing waveforms, whereas high-frequency biphasic pulsing primarily produces bubbles through boiling of the medium. Degassing due to reduced gas solubility at elevated temperatures appears to contribute only marginally. They further observed that boiling-induced bubbles collapse rapidly, while those formed via electrochemical reactions or degassing exhibit longer lifetimes.

Since early clinical PFA studies, microbubbles have been occasionally observed on intracardiac echocardiography immediately after energy delivery.^63^ These microbubbles have raised concerns due to their association with gaseous microemboli, which may contribute to silent cerebral lesions and stroke. The extent to which these events are directly attributable to the delivered current remains uncertain. Procedural factors such as catheter exchanges and other pathways for air entry into the bloodstream are also likely to play a significant role.^64^ In this regard, however, it is worth noting that a recent study using transcranial Doppler to monitor microembolization detected a higher rate of microembolic signals during PFA than during high-power short-duration radiofrequency ablation, with the ablation phase identified as the most embolic stage of the procedure.^65^

Nevertheless, the occurrence of embolic events cannot be attributed solely to gaseous microemboli. Given that most PFA systems employ high-frequency biphasic waveforms, which appear to generate bubbles primarily through boiling that collapse rapidly, it is plausible that at least a proportion of these microemboli instead arise from thrombus formation by blood coagulation due to excessive local heating.

Prevention or minimization of microbubble formation represents an opportunity for optimization of PFA systems. Catheter design can be refined to minimize gas entrapment, avoid sharp electrode edges, and reduce regions of high current density, thereby limiting thermal hotspots and arcing.^66^ In parallel, waveform and energy delivery can be carefully tailored to achieve effective tissue ablation while minimizing bubble generation. Further optimization of duty cycles and delivered energy, together with the introduction of pauses between bursts or the increase of irrigation rates, may allow sufficient time for gas and heat dissipation and thereby reduce bubble formation.^65,67^

### Arcing during pulsed field ablation

Arcing can occur when a gas is exposed to an electric field of sufficient strength to cause gas ionization (plasma) and dielectric breakdown. A similar phenomenon can happen during PFA if the transmission of pulsed electric fields causes bubble formation.^68,69^ Presence of gas is a necessary element of arcing, though a true arc is a continuous discharge of plasma, whereas ‘sparking’ is a better term for the PFA phenomenon. Since ‘arcing’ is almost universally used to refer to sparking during PFA, the term ‘arcing’ is used here.

Arcing was a major factor in complications from direct current (DC) ablation, such as barotrauma to cardiac tissue, when investigated in the 1980s using extracardiac defibrillators connected to diagnostic catheters.^70,71^ Since the pulse width used in DC ablation is milliseconds in duration, the level of gas formation and arcing that could occur was beyond what is typical in modern PFA systems. Modern PFA systems utilize microsecond or nanosecond pulse widths, which can limit the size of bubble formation and the consequences of arcing. Still, arcing continues to be relevant for modern PFA, as high current density (affected by electrode size and spacing) and energy-dense waveforms can result in arcs. The presence of metallic parts, namely left atrial appendage occlusion (LAAO) devices, in close proximity to the ablation catheter can produce a distortion of the electric field leading to a localize current density amplification. A bench test showed a similar risk of local arcing for three commercial PFA systems only when the distance between the catheter and the device was ≤1 mm. Good practices should avoid direct contact between PFA catheter and LAAO devices.^72^ However, PFA generators today include complex circuitry to detect high current discharge and stop ablations if necessary or the ability to turn off electrodes that are too close together.^73^

### Pulsed electric field interactions with other implanted electrical devices

Patients undergoing pulsed field ablation for the treatment of cardiac arrhythmias often have chronically implanted electronic devices for the treatment of cardiac or other conditions.^74,75^ Since PFA involves transmission of high-strength electric fields inside the body, such fields could interact with implanted electronic devices by interfering with their function or causing damage. Physical proximity is generally the biggest factor in adverse interactions of implantable devices and high-strength pulsed electric fields, as the field strength drops rapidly with distance from PFA catheter electrodes (proportional to 1/r or 1/r^2^, depending on catheter and electrode geometry). The importance of proximity makes interactions with cardiac implantable electronic devices (CIEDs) of particular interest.

The most common CIEDs that can interact with PFA electric fields include pacemakers (with transvenous leads and leadless), implantable cardiac defibrillators, and cardiac resynchronization therapy defibrillators. Though PFA was initially thought to be benign in regard to interactions with such CIEDs,^76,77^ issues have been discovered as the number of patients treated with PFA has increased.^78^ Adverse interactions of PFA and CIEDs fall into the following categories:^76^

- CIED oversensing of PFA signals
- Damage to a CIED due to PFA therapy
- Temporary or permanent damage to tissue surrounding a CIED
- Induction of an arrhythmia
- Ineffective PFA therapy or arcing
- Dislodgment of leads or device

Due to the recognized risk, a Heart Rhythm Society/European Heart Rhythm Society scientific statement now lists CIED interactions as a PFA safety issue, noting that risks can be platform-specific due to differences in catheter design, waveform, and ablation vector.^79^

## Pulsed field ablation waveform design

Since the specific characteristics of a PFA waveform can significantly impact the safety, efficacy, and usability of PFA systems,^80^ it is the purpose of this section to discuss the practical effects of some of the most impactful waveform characteristics. *Table 2* summarizes the key parameters that define these waveforms.

**Table 2:** Main features of PFA waveforms

Feature	Definition	Remarks
Amplitude	Maximum magnitude of waveform variation from its baseline.	Amplitude may refer to the peak magnitude of voltage, current, or electric field, depending on the context.
Pulse	Short-duration variation in the waveform, typically characterized by a rapid change from baseline to amplitude, followed by a return to the baseline.	Pulses are most commonly monophasic or biphasic square pulses, although sinusoidal oscillations or exponential decays have also been used in electroporation.
Phase configuration	It describes whether the magnitude of the waveform stays on one side of the baseline or alternates between positive and negative values.	Two possible configurations: Monophasic: The magnitude deviates from the baseline in only one direction. The pulses are either entirely positive or entirely negative. Biphasic: The magnitude alternates between two opposite polarities. A single pulse consists of both a positive and a negative phase.
Biphasic pulse	A biphasic pulse is a type of pulse consisting of two consecutive phases with opposite polarities. The waveform first deviates from the baseline in one direction (positive or negative), then reverses to an equal or different magnitude in the opposite direction before returning to baseline.	Biphasic pulses are often used to balance charge delivery and minimize electrochemical issues.
Interphase pause	Brief interval between the two phases of a biphasic pulse, during which the magnitude returns to baseline before the second phase begins.	
Burst (or train, or packet)	A burst is a sequence of pulses delivered in rapid succession as a single group, separated from other groups by longer intervals.	
Repetition rate (or frequency)	The repetition rate is the frequency at which pulses or bursts are delivered, typically expressed in hertz (Hz).	*f*
Repetition period	Inverse of the repetition rate (or frequency). It is the time interval between the start of one pulse (or burst) and the start of the next pulse (or burst).	$T=\frac{1}{f}$
Duty cycle	The duty cycle is the fraction of time a waveform is active relative to the total time, usually expressed as a percentage.	The duty cycle is generally proportional to the delivered energy. Low duty cycle waveforms are used in PFA because they enable the delivery of high electric field magnitudes while minimizing thermal side effects.

### Monophasic vs. biphasic pulses

High voltage pulses are applied between opposing polarity electrodes during PFA to create a high-strength electric field in cardiac tissue. Such pulses can be applied in a monophasic (maintains the same polarity throughout the pulse) or biphasic (polarity is reversed during the pulse) morphology (*Figure 3*).

**Figure 3:**
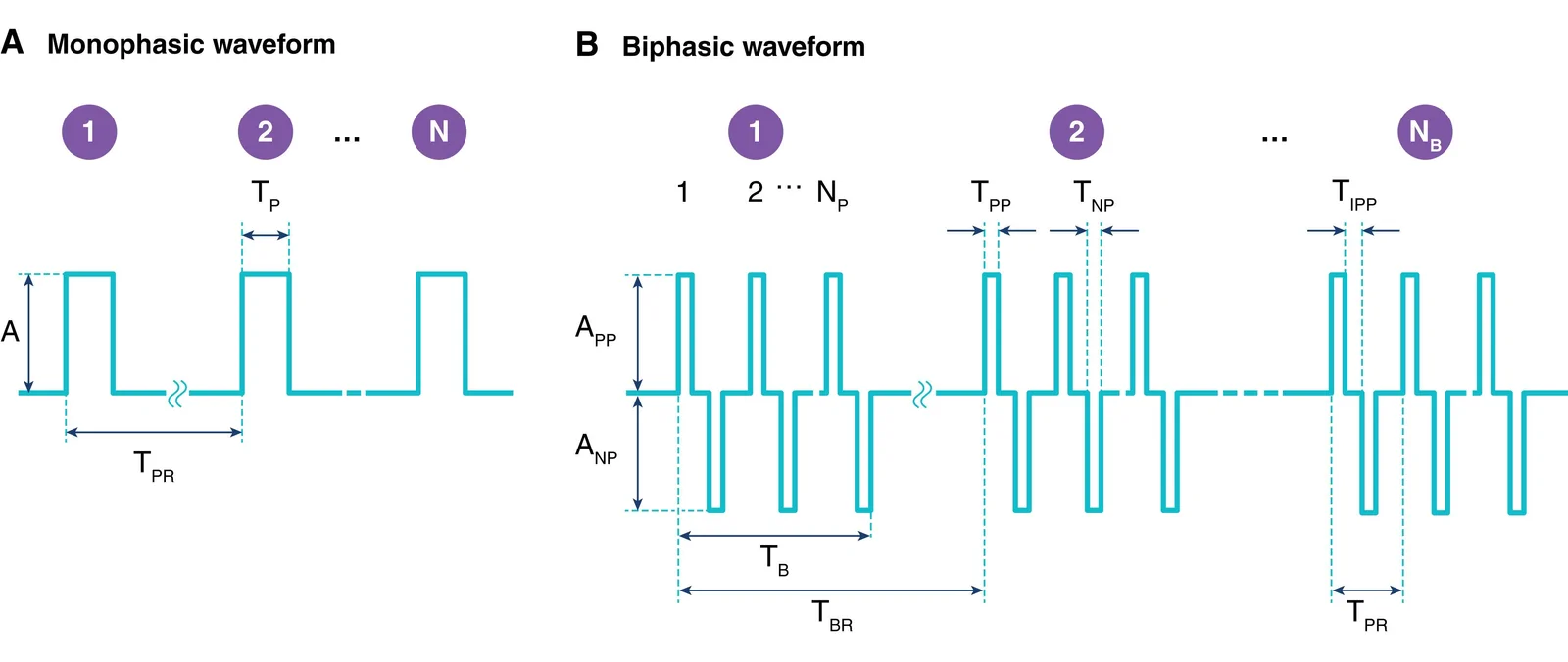
Main features of PFA waveforms composed of square pulses. *A*) Monophasic waveform: A = amplitude, T_P_ = pulse duration, T_PR_ = period of pulse repetition (i.e. 1/repetition rate), N = number of pulses. *B*) Biphasic waveform: A_PP_ = amplitude of the positive phase (of the biphasic pulses), A_NP_ = amplitude of the negative phase, T_PP_ = duration of the positive phase, T_NP_ = duration of the negative phase, T_IPP_ = duration of the interphase pause, T_PR_ = period of pulse repetition (i.e. 1/repetition rate), T_B_ = duration of the bursts (=trains = packets), T_BR_ = period of burst repetition, N_P_ = number of biphasic pulses in a burst, N_B_ = number of bursts.

Monophasic pulses have been shown to be more effective at creating cell damage than biphasic pulses for the same total energy,^13,81^ but they can also cause more undesired collateral effects.^68,82^ Biphasic pulses cause less musculoskeletal stimulation,^80,83^ less electrolytic bubble formation,^68^ and less risk of arrhythmogenicity due to cardiac stimulation,^84^ which is why most PFA systems utilize biphasic pulses.^79,85^

### Microsecond vs. nanosecond pulse widths

The duration of each pulse in a PFA waveform can cause cell damage via differing biophysical mechanisms. Pulse widths in the microseconds are hypothesized to induce a transmembrane voltage across the cell membrane, permeabilizing the cell via induction of pore formation and ion channel damage.^86^ Nanosecond pulse widths may cause more internal damage to the cell, bypassing the capacitive effects of the cellular membrane^85,87^ (*Figure 4*).

**Figure 4:**
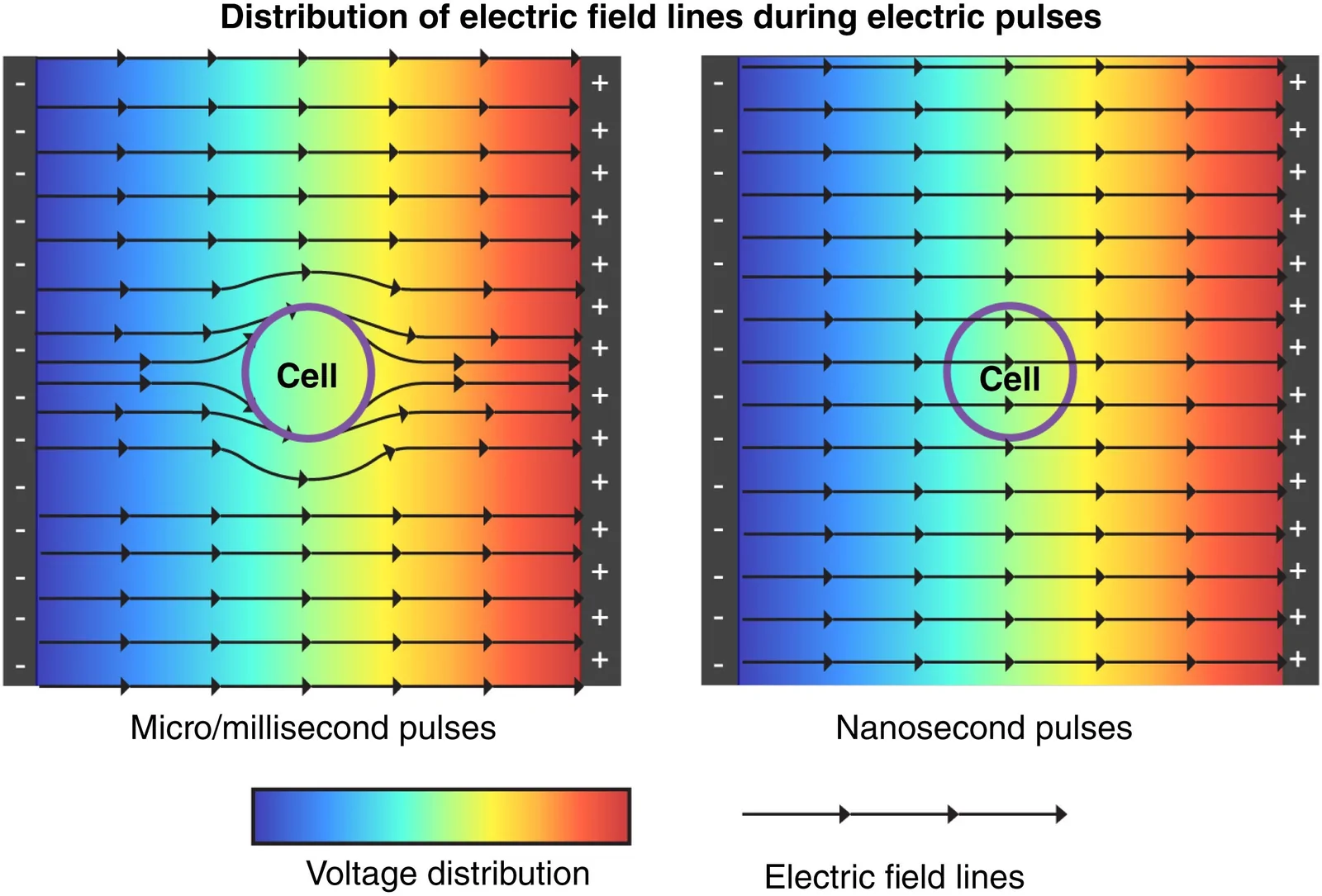
Differences in electric field flow around cells for microsecond and nanosecond pulses. Nanosecond pulses may bypass the capacitive effects of the cell membrane and cause more internal damage to the cell.

The selection of whether to use microsecond- or nanosecond-scale pulse widths depends on design goals for the PFA system across catheter, generator, patient anaesthesia, ablation dosing, and other factors. Of note, microsecond pulse widths can be produced with more straightforward generator hardware, as they require lower voltage for effective lesions (1–3 kV generally^84^). Nanosecond pulse widths require a higher voltage (8–12 kV generally) and thus require specialized generator designs,^88^ but may have advantages in ablation dosing and speed. Though many details of PFA waveforms remain trade secrets of PFA system manufacturers, the general scale of ‘microsecond’ or ‘nanosecond’ pulse widths is typically published.^84^ Many current microsecond-scale PFA systems use pulse widths in the low-microsecond range (roughly 1–10 ms),^89^ while nanosecond-scale PFA waveforms are more difficult to categorize. Nanosecond PFA waveforms could utilize pulse widths from single-digit nanoseconds up to hundreds of nanoseconds depending on manufacturer definition.

### Time duration and duty cycle of pulsed field ablation waveforms

Balancing competing goals in PFA waveform design also extends to total duration of each application and the distribution of pulses across the waveform. Pulsed field ablation applications can be shorter than those of thermal ablation modalities, with one commercially available PFA system utilizing 2.5 s per application as an example.^85^ Certainly, shorter ablation durations can allow faster procedures, though the number of ablations must be taken into account as well. The total duration of a PFA application is governed by the generator design (charging speed, voltage drop, etc.), the catheter design (number of electrodes, pulse vectors, catheter stability, heating near electrodes, etc.), and the biological environment (ability to capture tissue, proximity to sensitive structure, etc.). Pulsed field ablation waveforms are often delivered in bursts or trains of pulses that are repeated throughout the waveform, as opposed to a continuous pulse output. Pulse delivery in bursts can allow synchronization to the ventricular refractory period to reduce risk of arrhythmia induction,^90^ though most PFA in the atria is done in an unsynchronized fashion.^84^

## Pulsed field ablation catheter design considerations

An electric field is induced in living tissue when a voltage is applied across two electrodes. In the idealized case of a rectangular tissue volume sandwiched between two parallel plates, the electric field magnitude is simply the applied voltage divided by the electrode spacing. However, for other geometries—such as the catheters used in cardiac PFA—this relationship does not hold, and the electric field distribution must be determined via numerical methods (i.e. computer simulations). The electrode configurations are designed to maximize field magnitude and uniformity within the target region while minimizing localized hotspots. These high-intensity regions typically occur near electrode boundaries due to the edge effect. Additionally, material considerations such as electrode composition and surface roughness factor into the safety and efficacy of PFA application. Biocompatibility, electrochemical stability, corrosion resistance, and other factors typically lead to the use of platinum-iridium or gold instead of other materials like stainless steel for the electrode surface.^91^ In catheters with variable configurations or sizes instead of a fixed shape, the electric field dynamics must be investigated across the possible configurations.

Two main types of PFA catheter configurations can be distinguished: bipolar and monopolar configurations (*Figure* *5*). In the bipolar configuration, electrical energy is delivered between two closely spaced electrodes on the same catheter, resulting in a well-defined and confined electric field. In contrast, in the monopolar configuration, catheters deliver energy from electrodes at the catheter to a distant return electrode, typically a dispersive patch placed on the patient’s skin. A few PFA systems allow catheter configuration in either bipolar or monopolar mode but most commonly operate in a fixed configuration.

**Figure 5:**
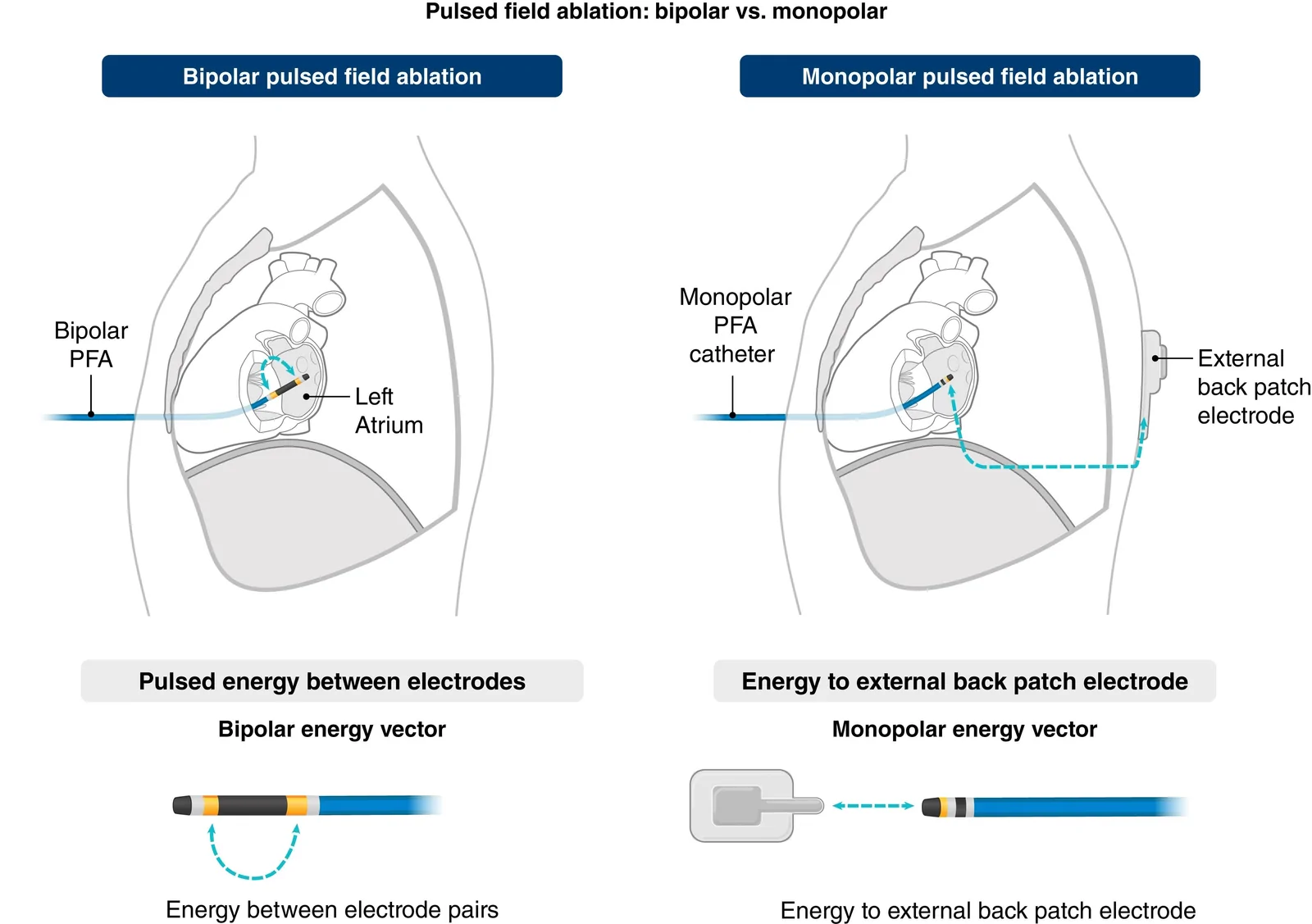
Differences in bipolar and monopolar energy vectors in pulsed field ablation. Bipolar ablations utilize electrode(s) on the catheter itself. Monopolar ablations send energy between electrode(s) on the catheter to the adhesive grounding pad, typically found on the back of the patient.

Because the return path in monopolar configurations is not well defined, it is often advantageous to control the delivered current rather than the voltage. Doing so helps reduce variability in the electric field strength near the catheter electrode, making it less sensitive to differing tissue and patch interface impedances in the return path.

In multi-electrode PFA catheters, whether operating in monopolar or bipolar configurations, energy is typically delivered sequentially to subsets of electrodes rather than simultaneously across the entire array. This approach reduces peak power demand and may enhance the uniformity and predictability of lesion formation by mitigating the effects of local tissue heterogeneities.^92^ Furthermore, the inclusion of additional electrodes within a bipole increases the effective surface area, thereby reducing the current density and associated risks.

### Clinical usability

Pulsed field ablation has always held the promise of increased patient safety as one of its primary appeals among clinicians. However, the possibility of simpler, faster, and more predictable ablation procedures also held appeal. Thermal ablation modalities, especially radiofrequency ablation, frequently required procedure durations of 1–2 h or longer, with poor predictability.^93^ The advent of PFA brought with it shorter procedure times and more predictability,^94^ important factors for optimizing caseload and managing lab staff. Beyond these positive procedural aspects of PFA, patient management has also become more straightforward. Patients complain less of chest pain and discomfort after a typical AF ablation with PFA, and the burden of patient follow-up is less for hospital staff.^95^ These are all positive factors that allow increasing the number of ablations completed per day and lessening the burden on lab staff.

Though the overall gains in procedure duration and lab management are important, there are usability aspects specific to each PFA system that must be considered, beginning with the most common ablation procedure, pulmonary vein isolation (PVI) for the treatment of AF. Usability here refers to the degree to which the full system—catheter, waveform, ablation workflow, and lab integration—facilitates intuitive, efficient, and reproduceable PFA procedures across diverse operators, anatomies, and clinical environments. The ideal for PVI has always been so-called ‘single-shot isolation,’ where each vein is permanently isolated from the rest of the left atrium by a single energy application. Though this could be accomplished with a cryoballoon, each ablation lasted minutes in duration.^90^ Perhaps an amended ideal of *rapid* single-shot isolation is required, and this has proven elusive, even for PFA. The most widely used PFA system across the globe, incorporating the pentaspline PFA catheter, prescribes a minimum of eight ablation applications per pulmonary vein for durable isolation. This ablation workflow includes catheter rotations and shape changes between ablations. Though such a workflow cannot be termed single-shot isolation, a widespread adoption of this technology serves as proof of the advantages of the ability to change catheter shape. Ablations can be form-fit to complex and varying patient anatomy, and the posterior wall ablation is increasingly being reported with ‘single-shot’ designs. On the other end of the spectrum are the spherical array or balloon-based PFA catheters, which can be used for true single-shot isolation for each pulmonary vein. However, this catheter form typically requires ablations of 40 s in duration^96^ and may not be as easily manoeuvrable and usable for other ablation targets as multispline catheters. Thus, trade-offs continue to be part of selecting a PFA system, at least until future generations are developed that can eliminate these trade-offs.

As PFA system designs continue to mature, continuously improving in safety and efficacy, it is possible that usability may be the defining difference between them. For instance, it is possible that the most usable systems of the future will be the ones able to accomplish the most tasks without catheter exchanges. One system and one catheter that can do complex electroanatomical mapping, single-shot PVI, posterior wall ablation, focal ablation, and lines of block in both atria would be a powerful tool. Ventricular mapping and ablation will also only grow in importance. And we must never forget that each geography around the world has different requirements, including in anaesthesia and procedural workflows. The most usable PFA systems may also be the ones that accommodate the most workflows and patient management options around the globe.

## Data Availability

The data underlying this article will be shared on reasonable request to the corresponding author.
